# Research on Blade Asynchronous Vibration Parameter Identification for Large-Scale Centrifugal Compressor Based on Improved MUSIC Algorithm

**DOI:** 10.3390/s25175351

**Published:** 2025-08-29

**Authors:** Zhenfang Fan, Yongtao Shen, Yupeng Du, Jinying Huang, Siyuan Liu

**Affiliations:** 1 School of Mechanical Engineering, North University of China, Taiyuan 030051, China; 2 School of Data Science and Technology, North University of China, Taiyuan 030051, China

**Keywords:** blade tip timing, blade asynchronous vibration parameter identification, multiple signal classification, estimating signal parameter via rotational invariance techniques

## Abstract

Blade tip timing (BTT) is a core technique for investigating the blade vibration of large-scale centrifugal compressors, and identifying the parameters of blade asynchronous vibration is crucial for implementing blade condition monitoring based on the BTT technique. In this study, the multiple signal classification (MUSIC) algorithm and the estimating signal parameters via rotational invariance techniques (ESPRIT) algorithm were first applied separately to identify the asynchronous vibration parameters of centrifugal compressor blades, with their advantages and disadvantages discussed. Subsequently, based on the frequency distribution characteristics in the ESPRIT results, the concept of “frequency distribution rate” was proposed. Finally, the results of the MUSIC algorithm were weighted by the frequency distribution rate, and an improved MUSIC algorithm was proposed. This enhanced confidence in the real frequency in the MUSIC algorithm results. Compared with the strain gauge method, the maximum relative error of the improved algorithm is 0.23%. The improved MUSIC algorithm improves the accuracy of parameter identification for blade asynchronous vibration, which holds great significance for the industrial application of the BTT technique.

## 1. Introduction

Centrifugal compressors, boasting advantages such as a high single-stage pressure ratio, a wide operating range, and a compact structure, have garnered significant attention and found critical applications in high-end manufacturing sectors including petrochemicals, natural gas, coal chemicals, and aerospace. The impeller serves as the core component responsible for energy conversion within a centrifugal compressor. However, due to the long working time, complex working conditions and harsh environment of the compressor, it is prone to cause vibration of the blades, resulting in local stress concentration on the blade surface and ultimately triggering fatigue failure [1]. Blade failure jeopardizes the operational safety of centrifugal compressors, imposing substantial economic losses and negative social impacts on enterprises. Consequently, blade vibration monitoring and vibration parameter identification are of great significance for the development and safe operation of centrifugal compressors [2].

Currently, scholars worldwide primarily focus on two methods for blade vibration monitoring: the strain gauge (SG) method and blade tip timing (BTT) technology. The former monitors blade vibration by attaching strain gauges, while the latter detects blade vibration using BTT probes. In practical applications, the SG method exhibits drawbacks such as limited measurement points, difficult signal transmission, and a short service life of strain gauges, failing to effectively address the issue of real-time monitoring of blade vibration in centrifugal compressors. In contrast, BTT technology enables continuous measurement of vibration signals from all blades and features non-contact measurement, providing reliable technical support for vibration monitoring of centrifugal compressor blades [3]. Blade vibration parameter identification involves extracting the characteristic parameters of blade vibration, including amplitude, phase, frequency, damping, etc., through the analysis of the undersampled BTT signal, which is the foundation for studying blade vibration problems. According to the principle of BTT technology, a single BTT probe measures each blade once per revolution, so the sampling frequency of a single BTT probe is approximately equal to the rotor’s rotation frequency. In practical applications, the blade vibration frequency of centrifugal compressors is much higher than the rotor’s rotation frequency, meaning the blade vibration signal based on BTT technology does not meet the Nyquist sampling theorem for frequency identification, and the blade vibration frequency obtained via BTT technology exhibits severe undersampling characteristics.

Blade vibration can be categorized into synchronous vibration and asynchronous vibration. Synchronous vibration refers to the blade vibration frequency being an integer multiple of the rotation frequency, while asynchronous vibration refers to the blade vibration frequency being a non-integer multiple of the rotation frequency. This study focuses on researching the parameter identification of blade asynchronous vibration. Scholars have carried out extensive studies on the algorithms for identifying blade asynchronous vibration parameters, considering factors such as the number of BTT probes and the layout of BTT probes. The algorithms for identifying blade asynchronous vibration parameters can be divided into four types: Fourier transform-based algorithms, eigenvalue decomposition-based algorithms, compressive sensing-based algorithms, and other algorithms.

In view of the non-uniformity and undersampling properties of BTT signals, scholars have improved the traditional Fourier transform algorithm. Vercoutter et al. [4] put forward a pseudo-spectral frequency identification algorithm based on multi-sampling averaging theory. Kharyton et al. [5] analyzed the vibration state of gas turbines during operation using a non-uniform Fourier transform algorithm. Carassale et al. [6] proposed an algorithm for identifying blade asynchronous vibration parameters at a constant speed, using a harmonic matching algorithm to identify single harmonic components and independent component analysis (ICA) to identify multiple harmonic components.

The principle of eigenvalue decomposition-based algorithms for identifying blade asynchronous vibration frequencies is to decompose the eigenvalues of the correlation matrix of blade vibration signals and identify the blade asynchronous vibration frequency by leveraging the relationships among the signal subspace, noise subspace, and eigenvectors. Eigenvalue decomposition-based algorithms include estimating signal parameters via rotational invariance techniques (ESPRIT) and multiple signal classification (MUSIC). The ESPRIT algorithm forms delayed and non-delayed channels using two BTT probes and identifies the blade asynchronous vibration frequency based on the phase difference between these two channels. The MUSIC algorithm identifies the blade asynchronous vibration frequency through spectral peak searching, utilizing the orthogonality between the signal vector and the noise subspace. Li et al. [7] used the ESPRIT algorithm to identify the blade asynchronous vibration frequency. Subsequently, He et al. [8] considered noise interference in actual experiments and proposed an ESPRIT algorithm based on the total least squares criterion (TLS), which improved the anti-interference ability of the ESPRIT algorithm. Li et al. [9] proposed an ESPRIT-based dual-probe method for monitoring blade vibration frequencies, achieving the time-frequency result of blade vibration with only two probes. Wang et al. [10,11] improved the traditional MUSIC algorithm, addressing the issue of limited frequency component recognition in the MUSIC algorithm and reducing its running time by reducing the dimensionality of the noise subspace. Later, Wang et al. [12] proposed adaptive subspace separation (ASS) and local spectral centroid (LSC) methods to enhance the adaptability of subspace selection and the stability of frequency identification, respectively. Liu et al. [13] discussed the reconstruction conditions of the MUSIC algorithm. Cao et al. [14] proposed an enhanced matrix completion technique (EMCT) for BTT signal post-processing and extracted frequency and amplitude parameters using the root-MUSIC and least square algorithms.

Compressive sensing-based algorithms sparsely represent the undersampled signals obtained by BTT technology, preserve the original structure of the signals through non-adaptive linear projection, and then accurately reconstruct the original signals through optimization problems, enabling parameter identification of undersampled signals with a sampling frequency much lower than the Nyquist sampling frequency. Chen et al. [15,16] proposed a compressive sensing-based order analysis method, and a compressive sensing method based on multi-coset angular sampling, respectively. Dong et al. [17,18,19] discussed optimization methods for different characteristics of the multi-coset sampling model, and improved the accuracy of blade asynchronous vibration parameter identification. Tian et al. [20] established a sparse reconstruction model for blade vibration signals under arbitrary circumferential arrangements of BTT probes. Subsequently, Spada and Nicoletti [21] optimized the number and layout of BTT probes as well as the number of signal points. Huang et al. [22] proposed a non-convex regularization-based undersampled sparse reconstruction method integrating displacement and velocity information.

Existing research also determines blade vibration parameters based on characteristics such as the original BTT signal and vibration frequency aliasing. Heller et al. [23] used capacitive probes to record the blade vibration waveforms, and proposed a global optimization-based blade vibration parameters identification method. Cao et al. [24,25] combined the achieved aliasing and the closed robust Chinese remainder theorem (CRT) to identify blade vibration parameters. Later, based on adaptive window short-time Fourier transform theory, a sampling-aliasing frequency (SAFE) mapping model-based blade resonance orders identification method was proposed.

This study focuses on the parameter identification problem of centrifugal compressor blade asynchronous vibration. The MUSIC algorithm and ESPRIT algorithm are used separately to identify blade asynchronous vibration parameters, and the advantages and disadvantages of these two algorithms in practical engineering applications are discussed. Subsequently, based on the ESPRIT algorithm, the concept of blade vibration frequency distribution rate is proposed, and the frequency distribution rate is used to improve the MUSIC algorithm results. The improved MUSIC algorithm shows excellent performance in identifying the frequency of centrifugal compressor blade asynchronous vibration.

## 2. Experiments

### 2.1. Blade Vibration Test of Large-Scale Centrifugal Compressor

**Figure 1:**
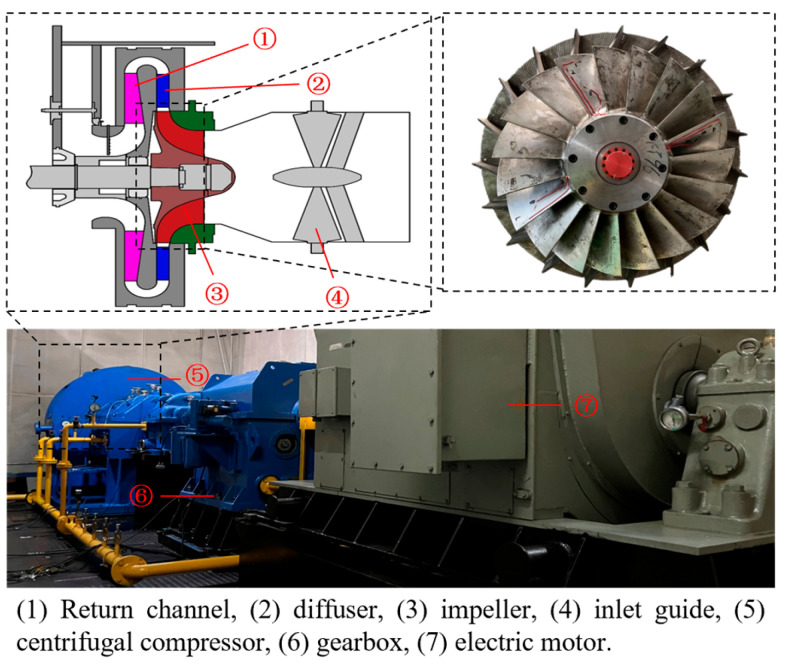
The Φ800 large-scale centrifugal compressor test rig.

Blade vibration tests were conducted on a centrifugal compressor test rig. The structural schematic diagram and physical diagram of the test rig are shown in Figure 1. The test rig includes an inlet guide vane, a centrifugal impeller, a diffuser, a return channel, etc. Two BTT probes were installed on the inlet ring of the centrifugal compressor at an angle of 10°. Strain gauges were attached to the root of the impeller blades for blade vibration testing, and the sampling frequency of the strain signals is 10,240 Hz. Detailed descriptions of the centrifugal compressor blade vibration test can be found in [26], which introduced a blade synchronous vibration parameters identification algorithm under variable speed conditions. This paper explores a blade asynchronous vibration parameters identification algorithm under constant speed conditions.

**Figure 2:**
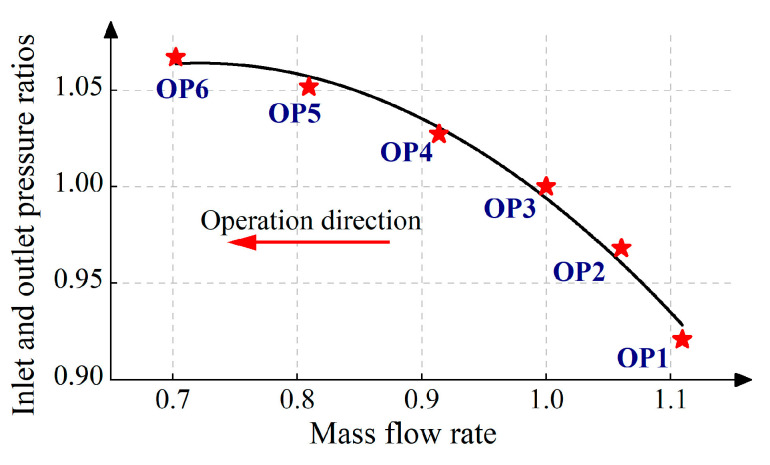
Test operating conditions of centrifugal compressor.

Under constant speed conditions, after the operating state of the centrifugal compressor stabilizes, the downstream throttle valve is gradually closed to adjust the flow rate of the centrifugal compressor, thereby bringing the centrifugal compressor into a surge state. During this process, blade vibration tests of the centrifugal compressor were performed using BTT technology and the SG technology. In addition, the flow rate and pressure of the centrifugal compressor under different working conditions were recorded. The blade vibration test of the centrifugal compressor was carried out at a rotation speed of 4800 rpm. Based on the flow rate and pressure parameters of the compressor under different operating points, the curves for mass flow rate and inlet–outlet pressure ratio at different speeds are shown in Figure 2. The flow rate in Figure 2 is normalized with respect to the rated flow rate.

### 2.2. Blade Vibration Measurement Results of Large-Scale Centrifugal Compressor

**Figure 3:**
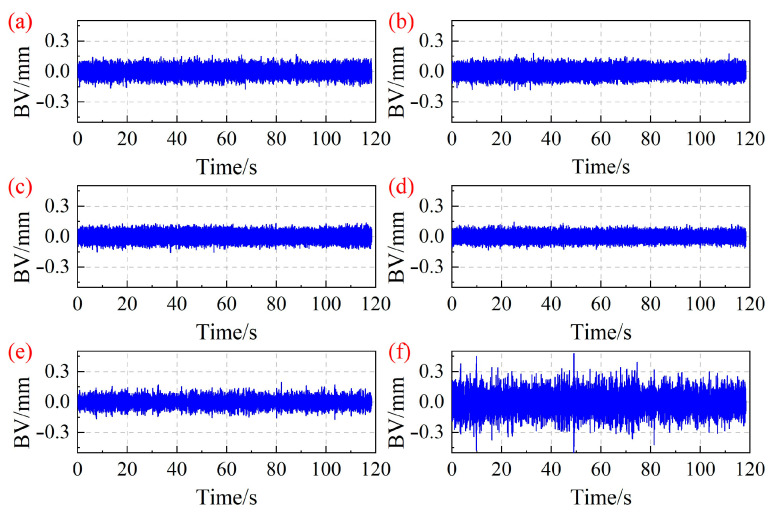
Vibration displacement results of the 7-th blade of the centrifugal compressor monitored by Tip1 under different operating conditions. (**a**) OP1; (**b**) OP2; (**c**) OP3; (**d**) OP4; (**e**) OP5; (**f**) OP6.

According to [27], two BTT probes can acquire four sets of signals, namely Tip1, Tip2, Tip3, and Tip4. Figure 3 shows the vibration displacement results of the 7-th blade of the centrifugal compressor monitored by Tip1 under different operating conditions. In Figure 3, the vertical axis title “BV” represents the blade vibration displacement, and the time length of the blade vibration signals is 118 s. It can be seen that under operating condition OP6, the blade vibration displacement increases remarkably, indicating that the centrifugal compressor is approaching the surge point [1].

## 3. Blade Asynchronous Vibration Parameters Identification Based on the MUSIC Algorithm

### 3.1. Theory Research

The MUSIC algorithm identifies vibration parameters through spectral peak searching by leveraging the orthogonality between the signal subspace and noise subspace of blade vibration. The following introduces the use of the MUSIC algorithm for identifying blade asynchronous vibration parameters. The blade asynchronous vibration signal can be characterized as a set of complex sinusoidal signals, and the expression of blade asynchronous vibration is as follows:

(1) $$y\left(t\right)=\sum_{k=1}^{K}a_{k}e^{2\pi f_{k}tj+\varphi_{k}}+v\left(t\right)$$

where *a*, *f*, and *φ* represent the blade vibration amplitude, frequency, and phase, respectively, *k* represents the blade vibration component, *j* represents an imaginary unit, and *ν*(*t*) represents white noise.

The BTT probe continuously collects *M* points of blade vibration signals, and the signal vector of the *q*-th sampling can be defined as follows:

(2) $$y\left(q\right)={\left[\begin{array}{cccc} y\left(t_{q}\right) & y\left(t_{q+1}\right) & \cdots & y\left(t_{q+M-1}\right) \end{array}\right]}^{T}$$

Ignoring the influence of noise during the measurement process and combining with (1), the blade vibration signal in (2) can be expanded as follows:

(3) $$y\left(q\right)=\left[\begin{array}{c} a_{1}e^{2\pi f_{1}t_{q}j+\varphi_{1}}+a_{2}e^{2\pi f_{2}t_{q}j+\varphi_{2}}+\cdots +a_{K}e^{2\pi f_{K}t_{q}j+\varphi_{K}}\\ a_{1}e^{2\pi f_{1}t_{q+1}j+\varphi_{1}}+a_{2}e^{2\pi f_{2}t_{q+1}j+\varphi_{2}}+\cdots +a_{K}e^{2\pi f_{K}t_{q+1}j+\varphi_{K}}\\ \vdots \\ a_{1}e^{2\pi f_{1}t_{q+M-1}j+\varphi_{1}}+a_{2}e^{2\pi f_{2}t_{q+M-1}j+\varphi_{2}}+\cdots +a_{K}e^{2\pi f_{K}t_{q+M-1}j+\varphi_{K}} \end{array}\right]$$

Considering the impact of noise, the blade vibration signal in (3) can be written in matrix form as follows:

(4) $$y\left(q\right)=As\left(q\right)+v\left(q\right)$$

where

(5) $$A=\left[\begin{array}{cccc} 1 & 1 & \cdots & 1\\ e^{2\pi f_{1}\tau_{1}j} & e^{2\pi f_{2}\tau_{1}j} & \cdots & e^{2\pi f_{K}\tau_{1}j}\\ \vdots & \vdots & \ddots & \vdots \\ e^{2\pi f_{1}\tau_{M-1}j} & e^{2\pi f_{2}\tau_{M-1}j} & \cdots & e^{2\pi f_{K}\tau_{M-1}j} \end{array}\right]$$

(6) $$s\left(q\right)=\left[\begin{array}{c} a_{1}e^{2\pi f_{1}t_{q}j+\varphi_{1}}\\ a_{2}e^{2\pi f_{2}t_{q}j+\varphi_{2}}\\ \vdots \\ a_{K}e^{2\pi f_{K}t_{q}j+\varphi_{K}} \end{array}\right]$$

(7) $$v\left(q\right)={\left[\begin{array}{cccc} v\left(q\right) & v\left(q+1\right) & \cdots & v\left(q+M-1\right) \end{array}\right]}^{T}$$

where

(8) $$\tau_{i}=t_{q+i}-t_{q}$$

Furthermore, matrix ***A*** is rewritten as follows:

(9) $$A=\left[\begin{array}{cccc} a\left(f_{1}\right) & a\left(f_{2}\right) & \cdots & a\left(f_{K}\right) \end{array}\right]$$

where

(10) $$a\left(f\right)={\left[\begin{array}{cccc} 1 & e^{2\pi f\tau_{1}j} & \cdots & e^{2\pi f\tau_{M-1}j} \end{array}\right]}^{T}$$

The autocorrelation matrix of the blade vibration signal ***y***(*q*) can be calculated as follows:

(11) $$R=E\left\{y\left(q\right)y^{H}\left(q\right)\right\}$$

*ν*(*t*) represents white noise with a mean of 0 and a variance of $\sigma_{v}^{2}$. Thus, the autocorrelation matrix ***R*** can be further expressed as follows:

(12) $$R=APA^{H}+\sigma_{v}^{2}E$$

Matrix ***P*** is a positive definite diagonal matrix:

(13) $$P=diag\left\{\begin{array}{cccc} {\left|a_{1}\right|}^{2} & {\left|a_{2}\right|}^{2} & \cdots & {\left|a_{K}\right|}^{2} \end{array}\right\}$$

It can be observed that matrix $APA^{H}$ has *K* non-zero eigenvalues. Perform eigenvalue decomposition on matrix $APA^{H}$, defining eigenvalues as $\lambda_{1}$, $\lambda_{2}$, ⋯, and $\lambda_{M}$, and the corresponding eigenvectors as $u_{1}$, $u_{2}$, ⋯, and $u_{M}$.

Therefore, the eigenvalues of the correlation matrix ***R*** can be derived as follows:

(14) $$\left\{\begin{array}{l} \begin{array}{ll} {\bar{\lambda }}_{i}=\lambda_{i}+\sigma_{v}^{2} & i=1,2,\cdots ,K \end{array}\\ \begin{array}{cc} {\bar{\lambda }}_{i}=\sigma_{v}^{2} & i=K+1,\cdots ,M \end{array} \end{array}\right.$$

The eigenvectors corresponding to the eigenvalues $\lambda_{1}$, $\lambda_{2}$, ⋯, and $\lambda_{K}$ are defined as the signal subspace, and the expression is as follows:

(15) $$E_{s}=\left[\begin{array}{cccc} u_{1} & u_{2} & \cdots & u_{K} \end{array}\right]$$

The eigenvectors corresponding to the eigenvalues $\lambda_{K+1}$, $\lambda_{K+2}$, ⋯, and $\lambda_{K+M}$ are defined as the noise subspace, and the expression is as follows:

(16) $$E_{n}=\left[\begin{array}{cccc} u_{K+1} & u_{K+2} & \cdots & u_{K+M} \end{array}\right]$$

According to the orthogonality between the signal subspace and noise subspace, when the blade vibration frequency is *f*, the following can be obtained:

(17) $$\left\{\begin{array}{c} {\left|a^{H}\left(f\right)u_{K+1}\right|}^{2}=0\\ {\left|a^{H}\left(f\right)u_{K+2}\right|}^{2}=0\\ \vdots \\ {\left|a^{H}\left(f\right)u_{M}\right|}^{2}=0 \end{array}\right.$$

By summing both sides of (17), the following can be obtained:

(18) $$\sum_{l=K+1}^{M}{\left|a^{H}\left(f\right)u_{l}\right|}^{2}=0$$

Moreover, (18) can be rewritten as follows:

(19) $$a^{H}\left(f\right)\sum_{l=K+1}^{M}u_{l}u_{l}^{H}a\left(f\right)=a^{H}\left(f\right)E_{n}E_{n}^{H}a\left(f\right)=0$$

In actual measurement, eigenvalue decomposition is performed by replacing ***R*** with the estimated $\overset{⌢}{R}$ of the correlation matrix of the blade vibration signal.

When the blade vibration frequency is *f*, the signal vector $a\left(f\right)$ and the noise subspace do not strictly satisfy the orthogonality condition. The right side of (19) is not equal to zero but rather a very small value. Therefore, the frequency search function can be constructed as follows:

(20) $$P_{m}\left(f\right)=\frac{1}{a^{H}\left(f\right)E_{n}E_{n}^{H}a\left(f\right)}$$

where $P_{m}$ is a dimensionless parameter, and its peak position reflects the estimation result of the blade vibration frequency.

Wang proposed an improved MUSIC algorithm [10], which is referred to as MR-MUSIC in this paper. In (2), the number of continuously sampled points *M* in the traditional MUSIC algorithm is equal to the number of BTT probes:

(21) $$M=N_{p}$$

where *N_p_* is the number of BTT probe. Nevertheless, the MR-MUSIC algorithm has been theoretically and experimentally proven to improve the identification accuracy of blade vibration parameters by increasing the value of *M*. Thus, the number of continuously collected points *M* is as follows:

(22) $$M=N_{R}N_{p}$$

where *N_R_* is the number of revolutions of the selected BTT signals.

### 3.2. Experiment Research

**Figure 4:**
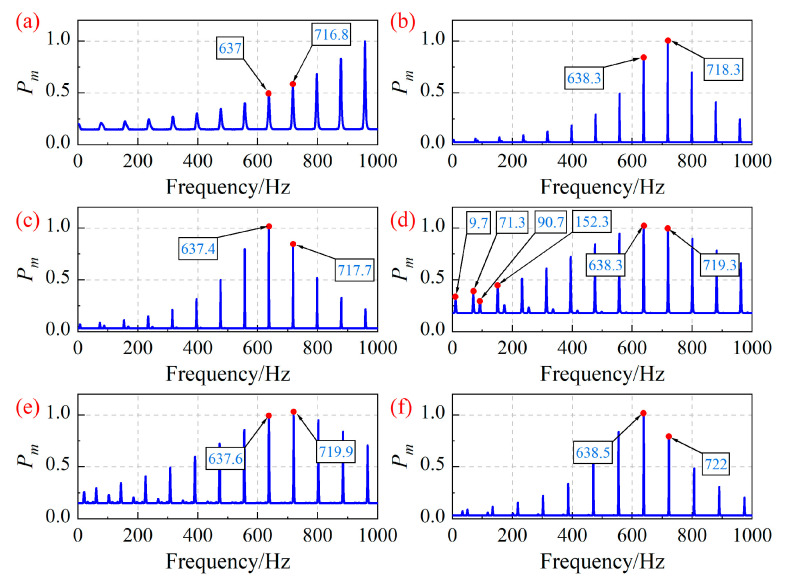
Asynchronous vibration frequency of the 7-th blade identified by the MR-MUSIC algorithm. (**a**) OP1; (**b**) OP2; (**c**) OP3; (**d**) OP4; (**e**) OP5; (**f**) OP6.

**Figure 5:**
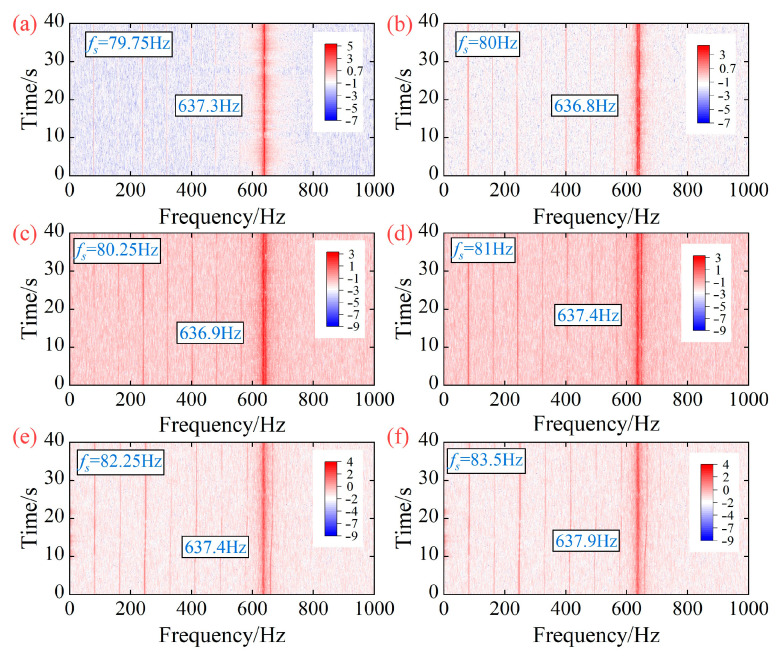
Time-frequency diagrams of the strain signal of the 7-th blade under different operating conditions. (**a**) OP1; (**b**) OP2; (**c**) OP3; (**d**) OP4; (**e**) OP5; (**f**) OP6.

Based on the blade vibration displacement shown in Figure 3, the blade asynchronous vibration frequency of the centrifugal compressor is identified using the MR-MUSIC algorithm. The number of revolutions of the selected BTT signals is set to 20. Figure 4 depicts the asynchronous vibration frequency of the 7-th blade identified by the MR-MUSIC algorithm. In addition, time-frequency analysis was performed on the strain signal of the 7-th blade. Figure 5 shows the time-frequency diagrams of the strain signal of the 7-th blade under different operating conditions. By comparing the blade vibration frequencies obtained through the MR-MUSIC algorithm and the SG method, the following conclusions can be drawn: (1) The MR-MUSIC algorithm can accurately identify the asynchronous vibration frequencies of the blades, as demonstrated in operating conditions OP3, OP4, and OP6; (2) when the impact of noise is significant, the confidence of the interference frequency is higher than that of the real frequency, making it challenging to accurately determine the blade vibration frequency in practical applications, such as in operating conditions OP1, OP2, and OP5.

## 4. Blade Asynchronous Vibration Parameters Identification Based on the ESPRIT Algorithm

### 4.1. Theory Research

Similar to the MUSIC algorithm, the ESPRIT algorithm constructs a correlation matrix of blade vibration signals, calculates the phase difference between two sets of blade vibration signals, and estimates the blade vibration frequency in combination with the delayed sampling time. In actual experiments, two BTT probes can form a delayed dual channel.

According to (2), the blade vibration signals measured by the two BTT probes at the *q*-th time are as follows:

(23) $$\left\{\begin{array}{c} y_{1}\left(q\right)={\left[\begin{array}{cccc} y_{1}\left(t_{q}\right) & y_{1}\left(t_{q+1}\right) & \cdots & y_{1}\left(t_{q+M-1}\right) \end{array}\right]}^{T}\\ y_{2}\left(q\right)={\left[\begin{array}{cccc} y_{2}\left(t_{q}\right) & y_{2}\left(t_{q+1}\right) & \cdots & y_{2}\left(t_{q+M-1}\right) \end{array}\right]}^{T} \end{array}\right.$$

Here, it is assumed that the installation angle between the two BTT probes is Δβ. Accordingly, the delay time between the two BTT probes is as follows:

(24) $$\tau^{†}=\frac{\Delta \beta }{2\pi f_{s}}$$

where $f_{s}$ means the rotation frequency in Hz.

Furthermore, (23) can be rewritten as follows:

(25) $$\left\{\begin{array}{l} y_{1}\left(q\right)={\left[\begin{array}{cccc} y_{1}\left(t_{q}\right) & y_{1}\left(t_{q+1}\right) & \cdots & y_{1}\left(t_{q+M-1}\right) \end{array}\right]}^{T}\\ y_{2}\left(q\right)={\left[\begin{array}{cccc} y_{1}\left(t_{q}+\tau^{†}\right) & y_{1}\left(t_{q+1}+\tau^{†}\right) & \cdots & y_{1}\left(t_{q+M-1}+\tau^{†}\right) \end{array}\right]}^{T} \end{array}\right.$$

According to (12), the vibration signals collected by the two BTT probes can be expressed as follows:

(26) $$\left\{\begin{array}{l} y_{1}\left(q\right)=\mathrm{As}\left(q\right)+v_{1}\left(q\right)\\ y_{2}\left(q\right)={A\Lambda }^{†}s\left(q\right)+v_{2}\left(q\right) \end{array}\right.$$

where

(27) $$\Lambda^{†}=\left[\begin{array}{cccc} e^{-2\pi f_{1}\tau^{†}j} & & & \\ & e^{-2\pi f_{2}\tau^{†}j} & & \\ & & \ddots & \\ & & & e^{-2\pi f_{K}\tau^{†}j} \end{array}\right]$$

Merge vectors $y_{1}\left(q\right)$ and $y_{2}\left(q\right)$ into one vector $Y\left(q\right)$, and the expression of $Y\left(q\right)$ is as follows:

(28) $$Y\left(q\right)=\left[\begin{array}{c} y_{1}\left(q\right)\\ y_{2}\left(q\right) \end{array}\right]=\left[\begin{array}{c} A\\ {A\Lambda }^{†} \end{array}\right]s\left(q\right)+\left[\begin{array}{c} v_{1}\left(q\right)\\ v_{2}\left(q\right) \end{array}\right]$$

According to (11) to (16), eigenvalue decomposition can be performed on vector $Y\left(q\right)$ to obtain the signal subspace $E_{s}$ and noise subspace $E_{N}$. Based on the time-invariant property of matrices, $E_{s}$ can be decomposed into two matrices, namely $E_{y_{1}}\in C^{M\times K}$ and $E_{y_{2}}\in C^{M\times K}$, and the relationship can be obtained as follows:

(29) $$E_{s}=\left[\begin{array}{c} E_{y_{1}}\\ E_{y_{2}} \end{array}\right]=\left[\begin{array}{c} \mathrm{AT}\\ {A\Lambda }^{†}T \end{array}\right]$$

Moreover, according to (29), there is:

(30) $$T^{-1}\Lambda^{†}T={\left(E_{y_{1}}^{H}E_{y_{1}}\right)}^{-1}E_{y_{1}}^{H}E_{y_{2}}$$

where the eigenvalues of ${\left(E_{y_{1}}^{H}E_{y_{1}}\right)}^{-1}E_{y_{1}}^{H}E_{y_{2}}$ is the diagonal element of $\Lambda^{†}$. By decomposing the eigenvalues of ${\left(E_{y_{1}}^{H}E_{y_{1}}\right)}^{-1}E_{y_{1}}^{H}E_{y_{2}}$ and calculating the phase carried by the eigenvalue $\begin{array}{cccc} \lambda_{1} & \lambda_{2} & \cdots & \lambda_{K} \end{array}$, the estimated value of blade asynchronous vibration frequency can be obtained as follows:

(31) $$P_{\mathrm{esprit}}\left(k\right)=\left|\frac{∠\lambda_{k}}{2\pi \tau^{†}}\right|$$

where $∠\left(\cdot \right)$ is the phase of the eigenvalue.

### 4.2. Experiment Research

Based on the blade vibration displacement shown in Figure 3, the blade asynchronous vibration frequency of the centrifugal compressor is identified using the ESPRIT algorithm. The data length of the blade vibration signal is set to 6000, and 60 data points are selected for each calculation, resulting in a total of 100 sets of results. To visually represent the results of the ESPRIT algorithm and assign values to each estimated frequency result, the following calculation formula is defined:

(32) $$\begin{array}{cc} P_{e}\left(i\right)=\frac{i}{100} & i=1,2,\cdots ,100 \end{array}$$

where *i* represents the *i*-th calculation by the ESPRIT algorithm, and $P_{e}\left(i\right)$ represents the amplitude corresponding to the *i*-th calculation, which is a dimensionless parameter.

**Figure 6:**
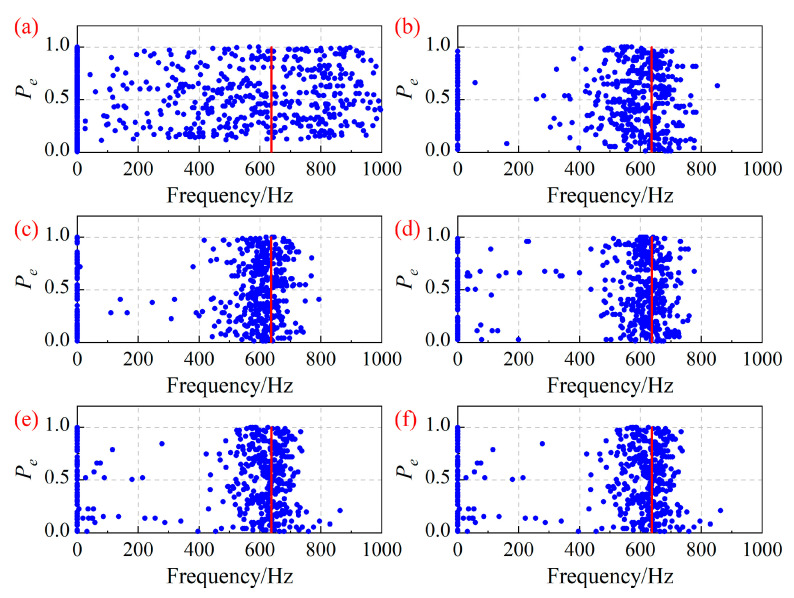
Asynchronous vibration frequency of the 7-th blade identified by the ESPRIT algorithm under different operating conditions. (**a**) OP1; (**b**) OP2; (**c**) OP3; (**d**) OP4; (**e**) OP5; (**f**) OP6.

Figure 6 shows the asynchronous vibration frequencies of the 7-th blade identified by the ESPRIT algorithm under different operating conditions. Here, the red lines represent the identification results obtained by the SG method. It can be concluded that the results identified by the ESPRIT algorithm are distributed around the real frequency, but the accuracy of frequency identification is relatively low.

## 5. Blade Asynchronous Vibration Parameters Identification Based on Improved MUSIC Algorithm

### 5.1. Theory Research

Figure 4 shows the identification result of the blade asynchronous vibration frequency by the MUSIC algorithm, and the main characteristic of the identification error is that the interference frequency is related to the half of the rotation frequency. Similarly, Figure 6 shows the identification results of the blade asynchronous vibration frequency by the ESPRIT algorithm, with the main feature of the identification error being a relatively large absolute error of the estimated frequency.

**Figure 7:**
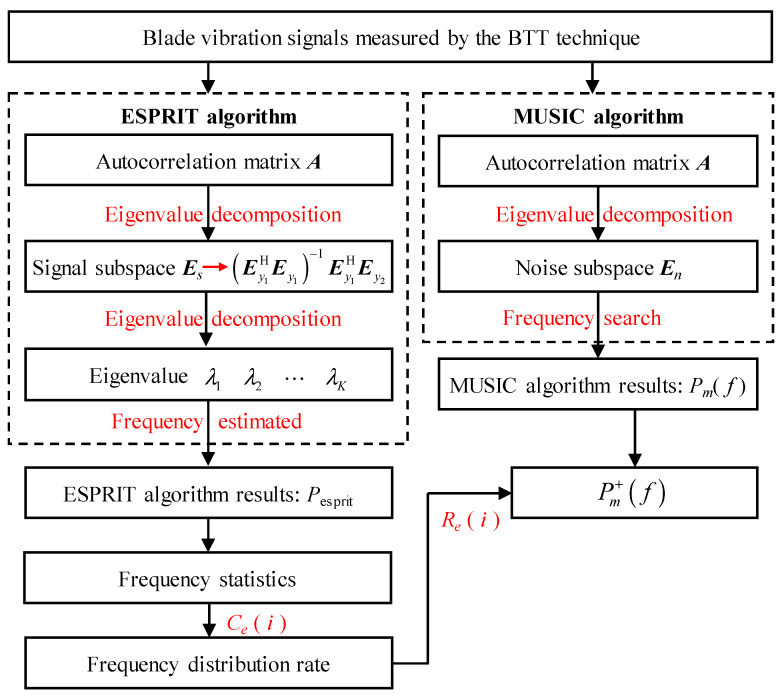
Flow chart of the improved MUSIC algorithm.

This study combines the advantages of the two algorithms and proposes an improved MUSIC algorithm for identifying the blade asynchronous vibration parameters of centrifugal compressors. First, the asynchronous vibration frequencies of the blade are identified by the MUSIC algorithm and the ESPRIT algorithm, respectively. Then, the frequency distribution rate of the identification results of the ESPRIT algorithm is calculated in different frequency bands. Finally, the confidence of the identified frequency in the MUSIC algorithm is enhanced through the frequency distribution rate of the ESPRIT algorithm. The improved MUSIC algorithm can reduce the influence of interference frequency and improve the identification accuracy of the MUSIC algorithm. The flowchart of the improved MUSIC algorithm is shown in Figure 7.

The expression of the statistical results of the ESPRIT algorithm and the corresponding frequency band are as follows:

(33) $$\begin{array}{cc} C_{e}\left(i\right)=Count\left(\begin{array}{cc} f_{s}/2*\left(i-1\right), & f_{s}/2*i \end{array}\right) & i=1,2,\cdots ,2N_{e} \end{array}$$

where Count(·) is the statistical quantity of ESPRIT algorithm results within the given frequency band range, and $N_{e}$ represents the identification range of frequency.

Furthermore, the frequency distribution rate of the ESPRIT algorithm can be obtained as follows:

(34) $$\begin{array}{cc} R_{e}\left(i\right)=\frac{C_{e}(i)}{\sum_{i=1}^{N_{e}}C_{e}(i)} & i=1,2,\cdots ,2N_{e} \end{array}$$

The frequency confidence in the MUSIC algorithm is enhanced by the frequency distribution rate in (34), and the calculation formula is as follows:

(35) $$\left\{\begin{array}{cc} P_{m}^{+}\left(f\right)=P_{m}\left(f\right)*R_{e}\left(1\right) & f\in \left[\begin{array}{cc} 0 & f_{s}/2 \end{array}\right]\\ P_{m}^{+}\left(f\right)=P_{m}\left(f\right)*R_{e}\left(2\right) & f\in \left[\begin{array}{cc} f_{s}/2 & f_{s} \end{array}\right]\\ \vdots & \vdots \\ P_{m}^{+}\left(f\right)=P_{m}\left(f\right)*R_{e}\left(2N_{e}\right) & f\in \left[\begin{array}{cc} f_{s}/2*\left(2N_{e}-1\right) & f_{s}N_{e} \end{array}\right] \end{array}\right.$$

### 5.2. Experiment Research

**Figure 8:**
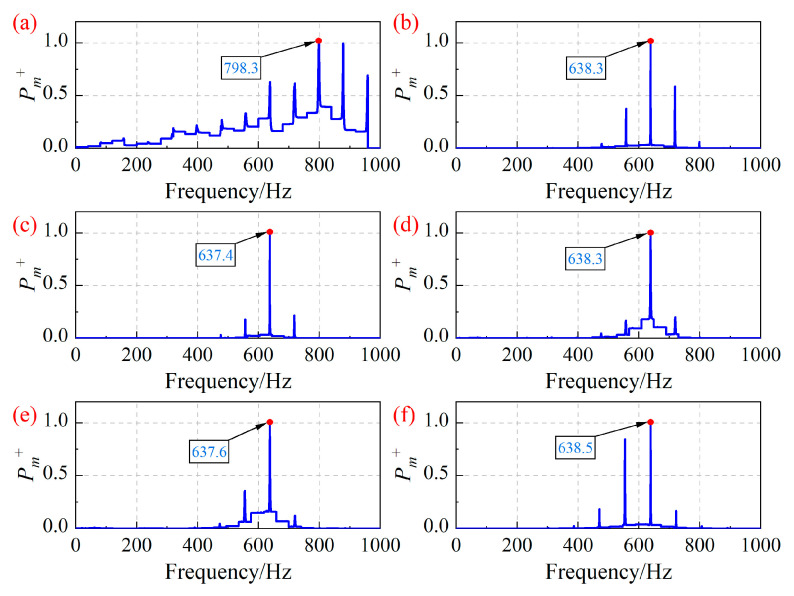
Asynchronous vibration frequency of the 7-th blade identified by the improved MUSIC algorithm under different operating conditions. (**a**) OP1; (**b**) OP2; (**c**) OP3; (**d**) OP4; (**e**) OP5; (**f**) OP6.

Based on the blade vibration displacement in Figure 3, the blade asynchronous vibration frequency of the centrifugal compressor is identified by the improved MUSIC algorithm, as shown in Figure 8. In Figure 8, the identification results of the blade asynchronous vibration frequency have been normalized based on the maximum value. Compared with Figure 4, the improved MUSIC algorithm can effectively reduce the influence of interference frequencies, and the identification accuracy of asynchronous vibration frequencies of blades is more significant. Figure 8a shows that the calculated results deviate significantly from the real frequency. This is because in actual experiments, there are fluctuations in the rotation speed. Under the operating condition OP1, the blade vibration frequency is approximately an integer multiple of the rotation frequency, indicating synchronous vibration of the blades. The MUSIC algorithm and the improved MUSIC algorithm still have limitations in dealing with the identification of blade synchronous vibration parameters.

Table 1 presents a comparison of the blade asynchronous vibration frequencies obtained by the SG method and the improved MUSIC algorithm. Furthermore, the relative error of the improved MUSIC algorithm can be calculated as follows:

(36) $$\mathrm{Relative}\;\mathrm{error}=\frac{\left|\mathrm{Improved}\;\mathrm{MUSIC}-\mathrm{SG}\;\mathrm{method}\right|}{\mathrm{SG}\;\mathrm{method}}\times 100\%$$

It can be concluded that compared with the SG method, the maximum relative error of the improved MUSIC algorithm is 0.23%, which meets the requirements of practical engineering applications.

## 6. Conclusions

This study aims to identify the asynchronous vibration parameters of centrifugal compressor blades. The MUSIC algorithm and the ESPRIT algorithm are used separately for identification, and the characteristics of the two algorithms in identifying blade asynchronous vibration parameters are discussed. Based on the results of the ESPRIT algorithm, the concept of the frequency distribution rate is proposed, and the frequency distribution rate is used to enhance the confidence of the real frequency in the MUSIC algorithm results, thereby proposing an improved MUSIC algorithm. The improved MUSIC algorithm effectively reduces the influence of interference frequencies and improves the identification accuracy of the blade asynchronous vibration parameters. However, this algorithm has not been validated in vibration signals with multiple frequency components, vibration signals at different speeds, and different BTT probe layouts, and further research is needed in future work.

## Figures and Tables

**Table 1:** Comparison of the asynchronous vibration frequencies of blades obtained by the SG method and the improved MUSIC algorithm.

Operating Condition	Improved MUSIC Algorithm	SG Method	Relative Error/%
OP2	638.3	636.8	0.23
OP3	637.4	636.9	0.08
OP4	638.3	637.4	0.14
OP5	637.6	637.4	0.03
OP6	638.5	637.9	0.09

## Data Availability

The raw data supporting the conclusions of this article will be made available by the authors on request.
